# Vaginal microbiome as a tool for prediction of chorioamnionitis in preterm labor: a pilot study

**DOI:** 10.1038/s41598-021-98587-4

**Published:** 2021-09-23

**Authors:** Daichi Urushiyama, Eriko Ohnishi, Wataru Suda, Masamitsu Kurakazu, Chihiro Kiyoshima, Toyofumi Hirakawa, Kohei Miyata, Fusanori Yotsumoto, Kazuki Nabeshima, Takashi Setoue, Shinichiro Nagamitsu, Masahira Hattori, Kenichiro Hata, Shingo Miyamoto

**Affiliations:** 1 Department of Obstetrics and Gynecology, Faculty of Medicine, Fukuoka University, 7-45-1 Nanakuma, Jonan-ku, Fukuoka, 814-0180 Japan; 2 Department of Maternal-Fetal Biology, National Research Institute for Child Health and Development, Tokyo, 157-8535 Japan; 3 Laboratory for Microbiome Sciences, RIKEN Center for Integrative Medical Sciences, Yokohama, 230-0045 Japan; 4 Department of Pathology, Fukuoka University School of Medicine and Hospital, Fukuoka, 814-0180 Japan; 5 Center for Maternal, Fetal and Neonatal Medicine, Fukuoka University Hospital, Fukuoka, 814-0180 Japan; 6 Department of Pediatrics, School of Medicine, Fukuoka University, Fukuoka, 814-0180 Japan; 7 Graduate School of Advanced Science and Engineering, Waseda University, Tokyo, 169-8555 Japan

**Keywords:** Diseases, Medical research, Pathogenesis, Risk factors

## Abstract

Intra-amniotic infection (IAI) is a major cause of preterm birth with a poor perinatal prognosis. We aimed to determine whether analyzing vaginal microbiota can evaluate the risk of chorioamnionitis (CAM) in preterm labor cases. Vaginal discharge samples were collected from 83 pregnant women admitted for preterm labor. Based on Blanc’s classification, the participants were divided into CAM (stage ≥ II; n = 46) and non-CAM (stage ≤ I; n = 37) groups. The 16S rDNA amplicons (V1–V2) from vaginal samples were sequenced and analyzed. Using a random forest algorithm, the bacterial species associated with CAM were identified, and a predictive CAM (PCAM) scoring method was developed. The α diversity was significantly higher in the CAM than in the non-CAM group (*P* < 0.001). The area under the curve was 0.849 (95% confidence interval 0.765–0.934) using the PCAM score. Among patients at < 35 weeks of gestation, the PCAM group (n = 22) had a significantly shorter extended gestational period than the non-PCAM group (n = 25; *P* = 0.022). Multivariate analysis revealed a significant difference in the frequency of developmental disorders in 3-year-old infants (PCAM, 28%, non-PCAM, 4%; *P* = 0.022). Analyzing vaginal microbiota can evaluate the risk of IAI. Future studies should establish appropriate interventions for IAI high-risk patients to improve perinatal prognosis.

## Introduction

Globally, approximately 15 million premature infants and 5–18% of pregnant women have experienced preterm birth^1,2^, which has been recognized as a contributing factor to perinatal mortality and morbidity. Additionally, preterm birth has been reported as the central cause of neurological damage in early childhood^3–5^ and remains the most common cause of infant mortality in middle- and high-income countries^2,6^. Intra-amniotic infection (IAI) is causally linked to preterm labor and accounts for approximately two-thirds of all preterm births^1,7^. Chorioamnionitis (CAM), indicated by inflammation of the fetal membranes during diagnosis, is the gold standard for corroborating IAI and is associated with preterm birth and poor infant prognosis, especially sepsis, chronic lung disease, cerebral palsy, and developmental disorders^8–17^. Bacteremias are identified in approximately 30% of all IAI cases^1,18^, and fetal infection causes a systemic inflammatory response^19^ that induces abnormalities in the central nervous system, as observed in animal experiments and epidemiological studies^20–22^. For a long time, attempts have been made to diagnose CAM and IAI before delivery^11,23–29^. However, no diagnostic standards have been established to date^11,27^. Therefore, the establishment of diagnostic and predictive methods for CAM is urgently needed in the field of perinatal care.

We previously demonstrated that it is possible to diagnose severe CAM with a high level of accuracy according to microbiomic CAM, defined by 16S rDNA amplicon sequence profiles and 16S rDNA copy numbers in the amniotic fluid^30^. Moreover, we found a large number of bacterial 16S rDNA copies in the amniotic fluid in cases of severe CAM, and the possibility of mixed infection by multiple bacterial species. Therefore, we expected to establish methods that can predict CAM using less invasive methods in the next step.

Bacterial vaginosis is associated with preterm birth^1,2,7,31,32^. Recently, including our previous report, many studies of amniotic fluid in cases with CAM, IAI, and/or preterm birth have found that many of the phlogogenic bacterial species in amniotic fluid might reside in the vaginal flora^30,32–38^. Although increases in dysbiosis and certain bacteria in the vaginal flora have been reported in cases of preterm birth and/or bacterial vaginosis, there are still many uncertainties regarding the diagnostic performance of different microbial markers determined using conventional methods, such as culture, polymerase chain reaction, general bacterial composition analysis using principal coordinate analysis (PCoA), and community state types (CST)^39–41^. However, it has been suggested that it might be possible to predict CAM based on the vaginal flora analysis. The prognostic value of vaginal flora could be investigated by using next-generation sequencing (an effective and comprehensive technique) and machine learning.

Our primary aim was to verify that analyzing the vaginal bacterial flora can help evaluate the risk of CAM. By narrowing the CAM-associated bacteria based on 16S rDNA amplicon sequencing data by general analysis and machine learning and creating an original scoring method, IAI with poor prognosis could be predicted.

## Results

### Assessment of participants

In total, 960 births occurred at the Center for Maternal, Fetal, and Neonatal Medicine, Fukuoka University Hospital, between May 2014 and April 2016. Placental pathology examination was conducted in 474 cases; among them, sampling of vaginal discharge was performed for 121 patients who were hospitalized with preterm labor. The sample collection was performed after the patients provided informed consent for participation. To eliminate the effects of bacterial contamination from the amniotic fluid, 38 cases of premature membrane rupture were excluded. Thus, 83 Japanese patients were included in the study.

Vaginal discharge samples were collected from 83 pregnant Japanese women admitted for preterm labor. Based on Blanc’s classification, which is used to classify the CAM stage^42^, a case–control study was conducted including two groups: the CAM (stage II or higher; n = 46) and the non-CAM group (stage I or no neutrophil infiltration [described as stage 0]; n = 37) (see Supplementary Table S1). The meta-data (characteristics, clinical data, laboratory data, and perinatal outcomes) of each group, extracted from medical records, are presented in Supplementary Table S2.

In this study, the gestational age at sampling was 30.4 ± 5.0 weeks (mean ± standard deviation) without a significant difference between the two groups (*P* = 0.118). Consistent with findings from previous studies, significant differences were observed in the maternal data regarding the history of arterial abortion, caesarean section, cervical length at sampling, heart rate at sampling, funisitis of the umbilical cord, and duration of hospital stay after birth. Moreover, neonatal data showed significant differences in the gestational age at birth, extremely preterm birth, neonatal body weight at birth, immunoglobulin M after birth, and chronic lung disease, in line with previous findings.

### Comparison of bacterial diversity

**Figure 1:**
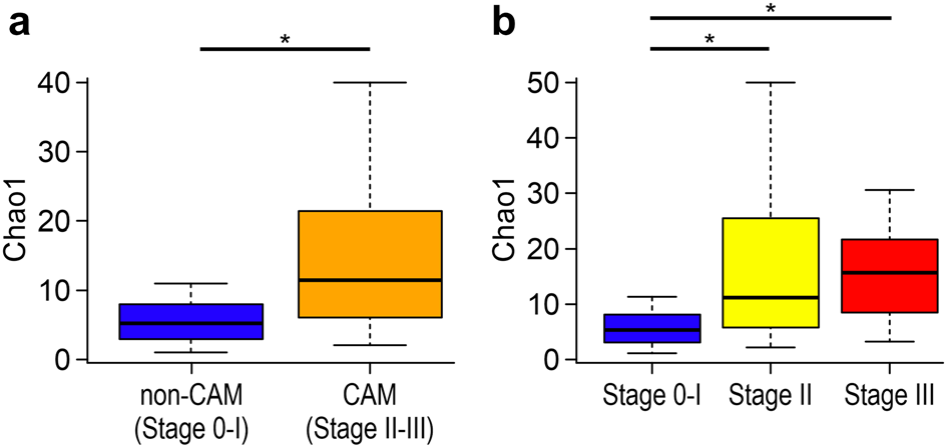
Chao1 indices. Sequences were clustered into operational taxonomic units with a 97% identity threshold, and the α diversity index (Chao1) was calculated for each sample. Chao1 was significantly higher in the CAM than in the non-CAM group. Chao1 was significantly lower in the stage 0–I group than in the other groups. Two-tailed probabilities were calculated by the Mann–Whitney test. *P < 0.05. *CAM* chorioamnionitis.

Similar to our previous study^30^, we used a universal primer set to target the V1–V2 region of the *16S rRNA* gene to sequence DNA extracted from the vaginal flora. The sequences were clustered into operational taxonomic units (OTUs) with a 97% identity threshold, and the within-community α diversity was assessed by comparing the α diversity index (Chao1) between the two groups (Fig. 1a). Interestingly, the median Chao1 value in the CAM group was 2.3 times that in the non-CAM group (median [interquartile range, IQR], 11.5 [6.3–21.5] and 5.0 [3.0–8.0], respectively; *P* < 0.001). The median Chao1 value in the severe CAM (stage III) group was 1.4 times that in the moderate CAM (stage II) group, although there were no significant differences between the groups (median [IQR], 15.5 [8.0–21.2] and 11.0 [5.5–25.0], respectively; *P* = 0.880) (Fig. 1b).

To compare the phylogenetic relatedness of the microbial communities in the β diversity analysis, UniFrac distances were determined between samples according to the OTU data. In the PCoA based on unweighted UniFrac distances, each group appeared to be scattered (see Supplementary Fig. S1). However, the distribution analysis based on the unweighted UniFrac distances using a permutational multivariate analysis of variance (PERMANOVA) revealed that the CAM group (stage II–III) was significantly different from the non-CAM group (stage 0–I) (*P* < 0.001), and both the CAM groups (stages II and III) were significantly different from the non-CAM group (*P* = 0.004 and 0.017, respectively); these findings were similar to the Chao1 results (see Supplementary Table S3). In the PCoA based on the weighted UniFrac distances, the samples were scattered without any clusters (see Supplementary Fig. S2). In the distribution analysis with PERMANOVA, the CAM group was not significantly different from the non-CAM group (*P* = 0.116), and both CAM groups (stages III and II) were not significantly different from the non-CAM group (*P* = 0.155 and 0.155, respectively) (see Supplementary Table S3).

### Analysis of bacterial composition in individual samples

Similar to our previous report^30^, phylum-, genus-, and species-level OTUs were created with identity thresholds of 70%, 94%, and 97%, respectively, and the taxonomic structure in each OTU was assessed by similarity searching against the standard database (see Supplementary Figs. S3–S5 and Supplementary Table S4). Moreover, the distribution of vaginal CSTs was also typed, similar to that reported by Ravel et al.^43^, and their occupancy rates in each group were compared (see Supplementary Fig. S6).

In many samples, *Lactobacillus* spp. (i.e., *L. iners*, *L. crispatus*, *L. gasseri*, *L. jensenni*) were dominant, and *Gardnerella* spp. (especially *Gardnerella vaginalis*) and *Prevotella* spp., which are known phlogogenic bacteria of bacterial vaginosis, and *Ureaplasma* spp. (especially *Ureaplasma parvum*), which were often reported to be associated with infectious preterm birth, were detected relatively frequently. No significant differences based on CAM and staging (Blanc’s classification) were observed in the compositions at the phylum, genus, or species level and frequencies of CSTs (see Supplementary Figs. S3–S6).

### Preparation of the scoring method based on the bacterial species associated with CAM

**Figure 2:**
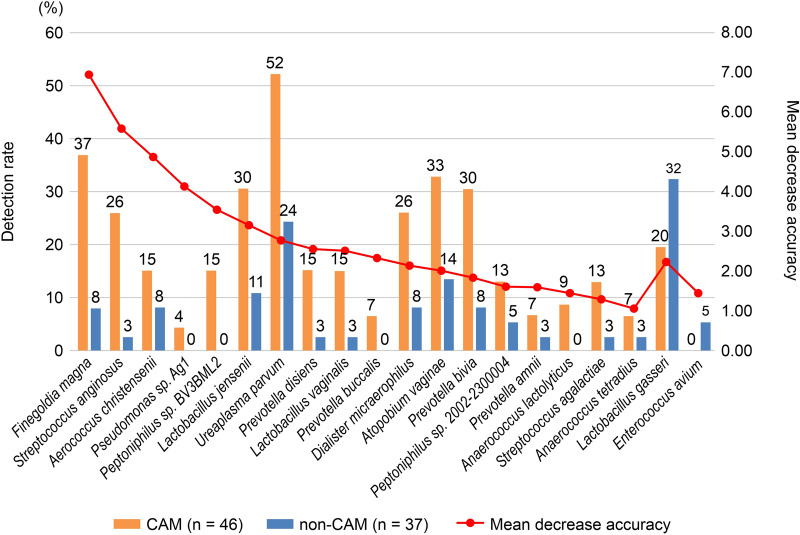
Detection rates of the 20 bacterial species most highly associated with CAM. The 20 bacterial species most highly associated (defined as a mean decrease accuracy > 1.0) with CAM were identified using the random forest algorithm. The bacterial species detected predominantly in the CAM group (orange) accounted for 18 out of 20 bacterial species (90%), whereas those in the non-CAM group (blue) accounted for only 2 out of 20 bacterial species (10%). The former was collected on the left and the latter on the right, and each bacterial species was sorted by the mean decrease accuracy. No significant difference was observed in any of the bacterial species. *CAM* chorioamnionitis.

Since the previous results in this study suggested that some bacteria with smaller amounts in the vaginal flora may contribute to the ascending infection into the uterus, the 20 bacterial species most strongly associated (defined as a mean decrease accuracy > 1.0) with CAM were identified using the random forest algorithm (see Supplementary Tables S4–S5 and Supplementary R Script online). The detection rates for each group are presented in Fig. 2. As expected, almost all 20 bacterial species were detected with a low composition ratio in each sample. The bacterial species detected predominantly in the CAM group accounted for 18 (90%) out of 20 bacterial species, whereas those in the non-CAM group accounted for only 2 (10%) out of 20 bacterial species. After adjusting for multiple testing using the Benjamin–Hochberg procedure, no significant difference was observed in any bacterial species.

**Figure 3:**
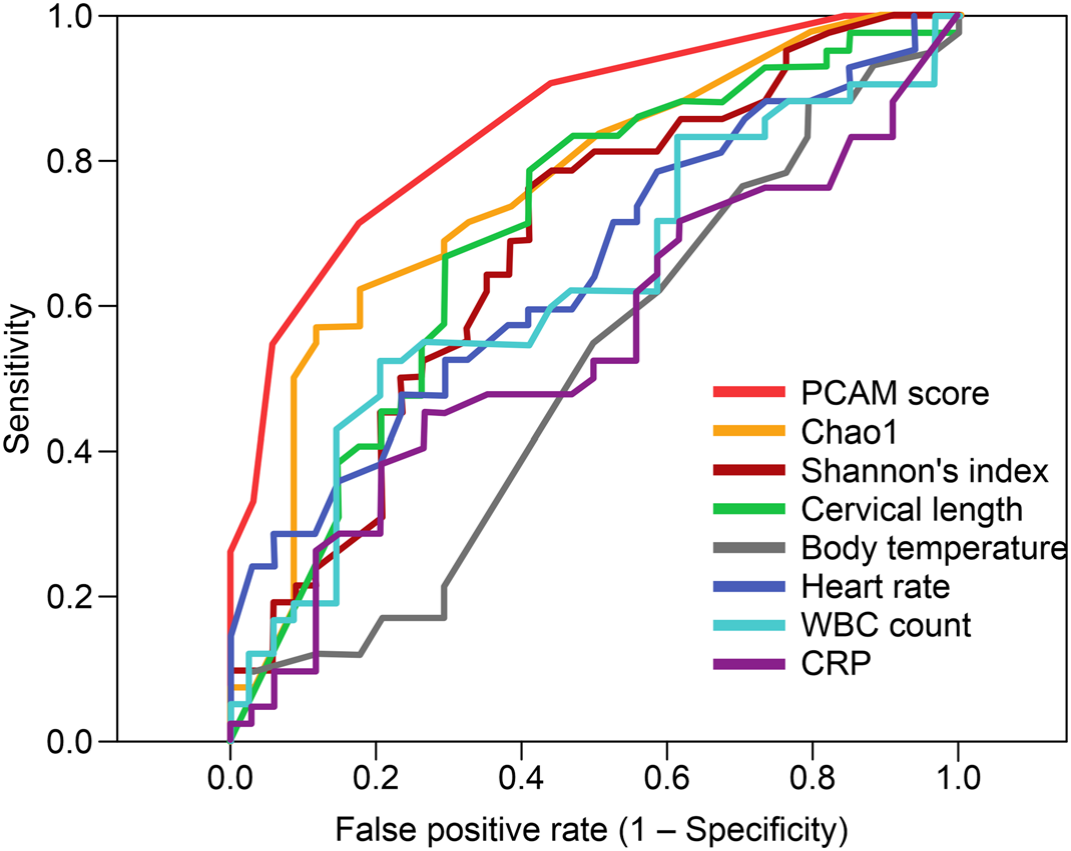
Predictive accuracy of PCAM scoring compared with the clinical data and α diversity indices. To compare the predictive diagnostic accuracy with the PCAM score and clinical indicators of body temperature, heart rate, white blood cell count, C-reactive protein value, cervical length, and α diversity index (Chao1, Shannon’s index), which comprised the common results of the 16S rDNA amplicon analysis, we calculated the AUCs using the ROC curve. The PCAM score was the most accurate measure, with an AUC of 0.849 (asymptotic 95% CI 0.765–0.934); sensitivity, 71.4%; and specificity, 82.4%. *AUC* area under the curve, *PCAM* predictive chorioamnionitis, *ROC* receiver operating characteristic.

**Figure 4:**
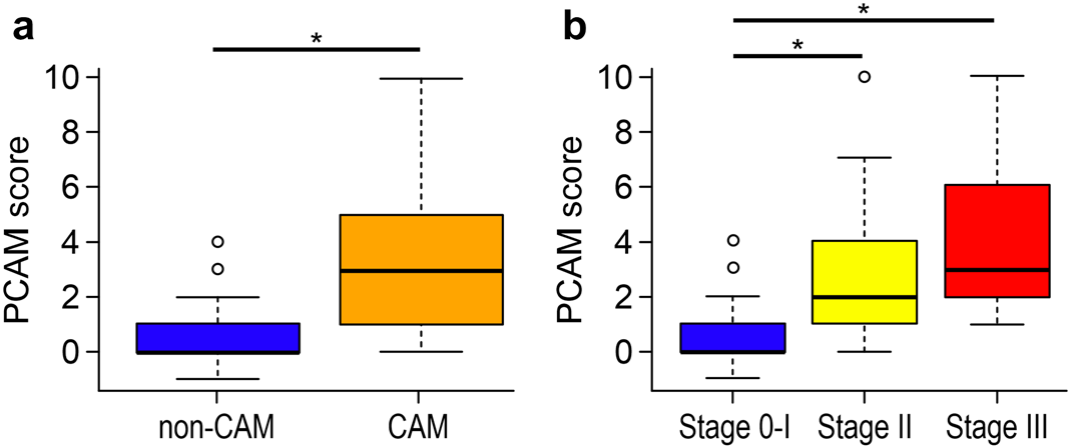
Comparing the PCAM scores. The PCAM score was calculated as the number of OTUs of the predominant bacteria in the non-CAM group subtracted from the number of OTUs of the predominant bacteria in the CAM group. The PCAM score was significantly higher in the CAM group (*P* < 0.001) and more clearly correlated with staging than the Chao1 indices. *P < 0.05. *CAM* chorioamnionitis, *OUT* operational taxonomic unit, *PCAM* predictive chorioamnionitis.

A scoring method was created using the OTU data of CAM-associated bacteria identified using the random forest algorithm. Considering the previously mentioned results of the α/β diversity analysis, the predictive CAM (PCAM) score was calculated as the number of OTUs of the predominant bacteria in the non-CAM group subtracted from the number of OTUs of the predominant bacteria in the CAM group. To compare the predictive diagnostic accuracy with the PCAM score and clinical indicators (i.e., body temperature, heart rate, white blood cell count, C-reactive protein value, cervical length, Chao1, and Shannon’s index), the area under the curve (AUC) was calculated using the receiver operating characteristic (ROC) curve for each item. The PCAM score had the highest predictive accuracy, with an AUC of 0.849 (asymptotic 95% confidence interval [CI] 0.765–0.934), sensitivity of 71.4%, and specificity of 82.4% (Fig. 3; see Supplementary Table S6). Moreover, the PCAM score was significantly higher in the CAM than in the non-CAM group (*P* < 0.001) and more clearly correlated with staging than the Chao1 indices (Fig. 4).

### Verification of clinical significance of the PCAM score

Since the cutoff value with the maximum Youden index in the PCAM score was 1.5 (see Supplementary Table S6), scores ≥ 2 and ≤ 1 were defined as PCAM-positive and PCAM-negative, respectively. In the subset analysis of the overall cohort, to eliminate bias caused by the clinical background, 47 cases of preterm labor at < 36 weeks of gestation, without hypertensive disorders of pregnancy, placental abruption, threatened rupture of the uterus, uterine malformation, intrauterine fetal death, or fetal chromosome abnormality, were divided into the PCAM (n = 22, PCAM-positive) and non-PCAM (n = 25, PCAM-negative) groups. The comparison results of the patient backgrounds are summarized in Supplementary Table S7, which are similar to the results presented in Supplementary Table S2. While verifying the predictive diagnostic accuracy of CAM, the PCAM score was found to be the highest, and the results for the AUC, Youden index, and cutoff value were almost the same with those previously reported (see Supplementary Tables S6 and S8).

The comparison between the PCAM and non-PCAM groups revealed no significant differences in the gestational age and weight at birth, but there was a significant difference in the pregnancy extension period from admission to birth. The median pregnancy extension period was approximately 2 weeks longer in the non-PCAM than in the PCAM group (median: PCAM group, 4 days; non-PCAM group, 17 days) (see Supplementary Table S9). The PCAM score was found to be the most useful criterion for diagnostic prediction. Moreover, it was also suggested that if we used CAM-associated bacteria in the PCAM group, as a therapeutic target, the gestation period would be extended by approximately 2 weeks. Moreover, although there was no statistically significant difference in Fisher’s exact test results (*P* = 0.07), the frequencies of developmental disorders at 3 years old were 28% (5/18 cases) and 4% (1/23 cases) in the PCAM and non-PCAM groups, respectively. Focusing on the six cases with developmental disorders at 3 years old in the sub-analysis, one case in the non-PCAM group was small for gestational age (SGA), but all five cases in the PCAM group were not SGA. A logistic regression analysis revealed a significant difference in developmental disorders at 3 years old between the two groups, adjusted by SGA (*P* = 0.022; odds ratio, 10.93; 95% CI 1.37–288.33; see Supplementary Table S10).

## Discussion

Many previous studies have suggested that IAIs, inducing preterm birth, fetal inflammatory response syndrome, and various sequelae and developmental disorders, are caused by the ascent of microbes into the uterus from the vaginal flora^1,2,7,17,31,32,44^. Recent studies investigating the vaginal microbiome have elucidated its relationship with preterm birth; however, many points are still unclear, particularly because of the possibility of racial and regional differences in the vaginal microbiome^2,43,45,46^. Therefore, we evaluated the status of IAI by the histological examination of the placenta, rather than by preterm birth along with various confounding factors, and revealed the association between histological CAM and the vaginal microbiota. Particularly, using machine learning and our simple scoring method, we developed a novel methodology that can be applied at multiple facilities. Furthermore, using data obtained from 3 years of follow-up after birth, we revealed that new prophylactic interventions against IAI can be expected to extend the gestational age and improve the fetal long-term prognosis.

Interestingly, the α diversity of the vaginal flora was significantly higher in the CAM than in the non-CAM group, and it was also revealed that the severity of CAM was correlated with a high α diversity. These results were consistent with findings from previous reports showing that the incidences of preterm premature rupture of membrane, IAI, and bacterial vaginosis as well as the α diversity in the vaginal flora in preterm births were relatively high^40,43,46–48^; these findings suggested that counting the bacterial species can be useful in assessing the risk of CAM. Furthermore, in the PCoA based on the UniFrac distance, the differences in the analysis of the unweighted UniFrac distance were higher than those on the weighted UniFrac distance. This result implied that specific bacteria associated with CAM were present in lower composition ratios (not predominant) in the vaginal flora of pregnant women, at least after the onset of preterm labor. This result was consistent with previous reports showing that a common microbiomic analysis could not identify specific bacterial characteristics in cases of preterm birth^49^.

Recent studies have reported that the diagnostic prediction accuracy was improved by using machine learning, such as the random forest algorithm, for the analysis of human microbiota^50–52^. We also used the random forest algorithm to extract bacterial species associated with CAM in the vaginal flora to improve reproducibility and objectivity. Moreover, based on the results of the α diversity analysis, we created a scoring method that simply counts the number of bacterial species that are associated with CAM in a patient. This novel scoring method improved the diagnostic prediction accuracy compared to various clinical findings of common microbiomic analysis (such as Chao1); no such attempt has been reported to predict IAI using this method.

Among the 20 CAM-associated bacterial species used for the new scoring method, 18 species were predominant in the CAM group and only two species were predominant in the non-CAM group, consistent with the results of the α diversity analysis in this study. Additionally, five bacterial species (*Streptococcus anginosus*, *Lactobacillus jensenii*, *U. parvum*, *Prevotella bivia*, and *Streptococcus agalactiae*) out of the 20 species are part of a group of 11 pathogenic bacterial species that are predominantly present in the amniotic fluid of patients with CAM^30^. Almost all of the other 15 species were also detected in the amniotic fluid in cases of preterm birth and/or premature rupture of membranes^38,38,53,54^. In particular, three species (*Finegoldia magna*, *Atopobium vaginae*, and *L. gasseri*) and genera (*Enterococcus* spp., *Peptoniphilus* spp. *Dialister* spp., and *Anaerococcus* spp.) were detected. Although *Ureaplasma* spp. in the amniotic fluid and vagina were reported to be associated with preterm birth and premature rupture of membranes^52–55^, they were also reported to be significantly associated with CAM in this study. *F. magna*, a species most closely associated with CAM, is a representative bacterial species of the gram-positive anaerobic cocci, mainly resident in the mucosa, and has been reported to be associated with soft tissue infections, bacterial vaginosis, and drug resistance to penicillin and clindamycin^55–57^. As aforementioned, *F. magna* is reported to be detected in the amniotic fluid in cases of preterm birth^53^; these findings, including our results, highlighted the requirement of further research on this topic. *Prevotella* spp. and *A. vaginae* are also vaginal bacteria that have been reported to be associated with preterm birth^2,45,46^. Interestingly, anaerobic bacteria, such as *Gardnerella* and *Mobiluncus* in the Nugent score of bacterial vaginosis, were not included among the 20 CAM-associated bacterial species. This was consistent with the fact that there was no significant difference in the frequency of CST IV between the CAM and non-CAM groups. Moreover, *Aerococcus christensenii* was reported to be detected in the amniotic fluid of patients with IAI^58^; however, further research is needed to confirm this finding.

As observed in the current results and in those from our previous study^30^, a specific vaginal microbiome may create an environment favorable for inducing the ascension of microbes into the uterus from the vaginal flora in patients with CAM. It is possible that increasing tolerability during pregnancy may not induce a simple infection but dysbiosis, such as quorum sensing in the vaginal flora, and cause an IAI, preterm birth, and the poor prognosis of infants. Similar to this study, a comprehensive analysis using next-generation sequencing is useful in analyzing specific vaginal dysbiosis. Furthermore, although regional and racial differences have been pointed out in the vaginal flora, it is possible to address this issue by slightly modifying the contents of machine learning for each facility.

To assess the clinical significance, we performed a subset analysis except for cases with a background that could induce a large bias directly linked to perinatal outcomes (such as preeclampsia). The results of the subset analysis reconfirmed the usefulness of this scoring, and indicated that if we correctly administer the appropriate interventions (i.e., antibiotics, prebiotics, and transplantation of bacterial flora) to the PCAM group in preterm labor cases, the extended period of pregnancy and poor prognosis of infants can be improved significantly. This hypothesis seemingly contradicts the fact that antibiotics cannot prevent preterm births; however, it has been suggested that many previous reports investigating this issue may have included patients with a low risk, without undergoing a sufficient risk assessment of IAI^59^. Moreover, although this study did not show the antibiotic administration history and did not indicate that IAI infection may have been induced by loss of typical microbiota (such as *Lactobacillus* spp.), it is clear that typical microbiota is inhibitory to IAI. Therefore, it may be necessary to be aware of the risk that antibiotics may induce preterm birth and IAI^60^. To prevent these issues, it may be better to combine antibiotic and prebiotic (such as lactoferrin) treatments, which have been reported to induce normal bacterial flora and/or normal bacterial flora transplantation^61,62^. Further, we should consider the clinical background, such as genetic factors and complications related to preterm birth. However, there are many unclear points regarding the relationship with tocolytic drugs and progesterone therapy. Therefore, further research on the selection of treatment methods is needed.

This study had some limitations. First, the sample size was small. Many previous microbiome studies, including our previous studies, have not focused on the long-term prognosis of infants, as it was thought that this would lengthen the study period and impair the associated novelty while waiting for it. However, we considered that the relationship with prognosis would be important and examined the prognosis up to the age of 3 years. Although there was no significant difference between the two groups in the frequency of developmental disorders at 3 years old in Fisher’s exact test, as adjusted by SGA using a multivariate analysis, a significant difference was revealed. In the five cases with developmental disorders at 3 years old in the PCAM group in the sub-analysis, the mean ± standard deviations of the gestational age and neonatal birth weight were 31.5 ± 4.9 weeks and 1597 ± 672 g, respectively; thus, the involvement of infection was strongly suspected. Future studies are needed to identify patterns of the vaginal flora that are associated with a particularly poor prognosis. Second, this was a pilot study conducted at a single institution. Since regional and racial differences in the vaginal microbiome have been reported, it is difficult to apply our findings to each facility^2,43,45,46^. However, we speculate that our scoring method, which narrows down the list of specific bacteria associated with CAM by machine learning and simply counts them, will be highly versatile. In addition to machine learning, such as the random forest algorithm, the use of artificial intelligence may be useful for narrowing down the CAM-associated bacteria, and future research is expected in this regard. Third, it is difficult to apply next-generation sequencing in clinical practice. As this was a retrospective study, it was possible to carry out high-quality sequencing using MiSeq (Illumina, San Diego, CA, USA), but the clinical application of this technology is difficult in terms of the cost and time required for a single analysis. However, in recent years, methods that can rapidly analyze the bacterial flora using the nanopore system have been reported, and if these methods can be applied, it may be possible to analyze each case at each institution and start the treatment promptly^63^. In addition, after validation has been performed in a larger cohort, this platform may be changed to clinically easy-to-use systems, such as multiplex polymerase chain reaction and loop-mediated isothermal amplification systems.

In this study, we comprehensively analyzed the vaginal microbiome and identified the bacteria significantly associated with CAM by examining its association with the degree of placental inflammation. If PCAM scoring is applied in clinical settings, the prediction of the subsequent IAI is possible during early pregnancy even if there are racial and regional differences in the vaginal microbiome. Future studies should focus on establishing appropriate interventions for IAI high-risk patients to improve perinatal prognosis.

## Methods

### Study design

As in our previous reports, histologic CAM was defined as the presence of acute inflammatory lesions of the decidua, chorion, or amnion according to Blanc’s criteria^29,30^. The pregnant women were divided into two groups based on Blanc’s classification of placental inflammation severity: the CAM (stage II–III) (n = 46) and non-CAM groups (stage I or no neutrophil infiltration) (n = 37) (see Supplementary Table S1).

All the methods were performed according to the STARD guidelines and regulations for reporting diagnostic accuracy studies. This study was approved by the review boards of Fukuoka University Hospital and the National Research Institute for Child Health and Development (no. 15-2-08 and 699, respectively). Informed consent was obtained from all patients who were informed about the potential risks, including accidental leaks of personal information and project data, prior to the study initiation. For participants who wish to withdraw the information obtained from them, we can dispose of the remaining samples and project data; however, we cannot delete the sequence data that have been made publicly available through an open-access database.

### Diagnostic criteria

As in our previous report^30^, CAM was histologically defined as the presence of acute inflammatory lesions of the chorion or amnion according to Blanc’s criteria^42^ as follows: stage I (sub-chorionitis), patchy or diffused accumulation of neutrophils within the sub-chorionic plate or decidua; stage II (chorionitis), more than a few scattered neutrophilic infiltrations in the chorionic plate or membranous chorionic connective tissue; stage III (CAM), neutrophilic infiltrates reaching the sub-amniotic connective tissue and the amniotic epithelium. Funisitis was defined as a neutrophilic infiltration in the umbilical vein wall or Wharton’s jelly.

Similar to our previous report^30^, according to Lencki et al.^23^, clinical CAM was diagnosed if patients had fever ≥ 38 °C with no evident infection and at least one of the following signs or symptoms: maternal pulse ≥ 100 beats/min, uterine tenderness, malodorous vaginal discharge, or maternal white blood cell count ≥ 15,000/mm^3^. Premature rupture of the membranes was considered to have occurred if we observed evident outflow of weakly alkaline amniotic fluid or detected insulin-like growth factor-binding protein 1 in the vaginal discharge. All the cases of developmental disorders were diagnosed by a pediatric specialist according to the Kyoto Scale of Psychological Development, which is one of the most widely used developmental tests in Japan^64^.

### Sample collection, DNA extraction, and 16S rDNA amplicon sequencing

The participants’ vaginal discharge at the midpoint of the vagina was collected using Opti-Swab 1 mL liquid Amies transport medium sterile elongated flock swab (Puritan Medical Products, Guilford, ME, USA) by the placement of a sterilized vaginal speculum. Collected vaginal swab samples were kept at 4 °C, frozen in liquid nitrogen within 24 h after sampling, and preserved at − 80 °C until DNA extraction. Microbial DNA was isolated from the samples, and the 16S rDNA amplicon sequencing library was prepared according to a previously described method^30^. The 16S ribosomal RNA gene’s hypervariable V1–V2 regions were amplified by polymerase chain reaction using universal primers (27Fmod and 338R) and multiplexed. MiSeq sequencing (paired end, 300 bp) was performed using the MiSeq Reagent Kit v3 (600-cycle format; Illumina) mixed with 20% of the PhiX Control Kit v3 (Illumina) according to the manufacturer’s protocol.

### Analysis of sequencing data

As aforementioned^30,33^, sequences were assigned to samples based on their barcode sequence, and two paired-end reads were merged based on overlapping sequences. Low-quality sequence reads (quality value < 25) and suspected chimeric reads (alignment length of < 90% coverage with reference sequences in the Ribosomal Database Project version 10.27 and/or in-house 16S database) were filtered out (see Supplementary Table S11). After adapter trimming, 1500 reads per sample were randomly selected. The selected reads were clustered into OTUs using a 97% pairwise identity cutoff with the UCLUST program^65^ version 5.2.32 (http://www.drive5.com/). The representative sequences of the OTUs were blasted to the above-mentioned databases using the GLSEARCH program to determine the closest taxa. To assign OTUs to the taxa at the phylum, genus, and species levels, we applied sequence similarity thresholds of 70%, 94%, and 97%, respectively. The UniFrac distance was calculated to assess the dissimilarity (distance) between each sample^66^, and a PCoA was plotted according to the UniFrac distances.

Five types of vaginal bacterial CSTs were classified according to previous reports^43,67^. Four of these were dominated by *Lactobacillus* spp., including *L. crispatus* (CST I), *L. gasseri* (CST II), *L. iners* (CST III), and *L. jensenii* (CST V). CST IV was characterized by small relative abundances of *Lactobacillus* spp. and increased relative abundances of anaerobic bacteria, including *Prevotella*, *Dialister*, *A. vaginae*, *G. vaginalis*, *Megasphaera*, *Peptoniphilus*, *Sneathia*, *Finegoldia*, and *Mobiluncus*.

### Creating the novel scoring method

Similar to previous studies^68–70^, the bacterial species associated with CAM were identified based on OTU data using the random forest algorithm with default setting in R version 3.3.1 (R Foundation for Statistical Computing, Vienna, Austria; see the Supplementary R Script online). The degree of association was evaluated using the mean decrease accuracy. The 20 bacterial species most strongly associated (defined as mean decrease accuracy > 1.0) with CAM were identified, and the PCAM score was calculated as the number of OTUs of the predominant bacteria in the non-CAM group subtracted from the number of OTUs of the predominant bacteria in the CAM group.

### Statistical analysis

As described previously^30^, the normalities of distribution of the continuous variables were assessed using the Kolmogorov–Smirnov test, and the exact significance probabilities (two-tailed) in the form of *P*-values were calculated using the Mann–Whitney and Fisher’s exact tests for continuous and categorical variables, respectively. *P*-values were adjusted for multiple testing by the Benjamin–Hochberg procedure^71^. Random forest models were generated using the AUC-RF package^72^. ROC curves were constructed to assess the diagnostic and predictive accuracy. These analyses were performed using SPSS version 16.0 for Windows Base System SC (IBM Corp., Armonk, NY, USA). Multivariate analysis was performed using logistic regression models from the JMP software program, version 14 (SAS Institute, Cary, NC, USA). Then, the crude and multivariable *P*-values, odds ratios, and their 95% CIs were calculated. For the comparison of bacterial composition between groups, the vegan package in R version 3.3.1 was used to calculate the R^2^ and *P*-values in a PERMANOVA. Differences were considered statistically significant at *P* < 0.05.

## Supplementary Information

Supplementary Information.

Supplementary Table S4.

## Data Availability

The raw next-generation sequencing data with clinical data have been deposited in the DDBJ Sequence Read Archive (DRA) (accession number DRA011863).
