# Supplementary material for: Vaginal microbiome as a tool for prediction of chorioamnionitis in preterm labor: a pilot study
Source: Sci Rep. 2021 Sep 23;11:18971. doi: 10.1038/s41598-021-98587-4 (PMC8460623; doi:10.1038/s41598-021-98587-4)
Supplement: Supplementary file 1 — Supplementary Information. [file 41598_2021_98587_MOESM1_ESM.docx]

Supplementary Information

Vaginal microbiome as a tool for prediction of chorioamnionitis in preterm labor—a pilot study

Daichi Urushiyama, Eriko Ohnishi, Wataru Suda, Masamitsu Kurakazu, Chihiro Kiyoshima, Toyofumi Hirakawa, Kohei Miyata, Fusanori Yotsumoto, Kazuki Nabeshima, Takashi Setoue, Shinichiro Nagamitsu, Masahira Hattori, Kenichiro Hata, Shingo Miyamoto

**Supplementary Figures**


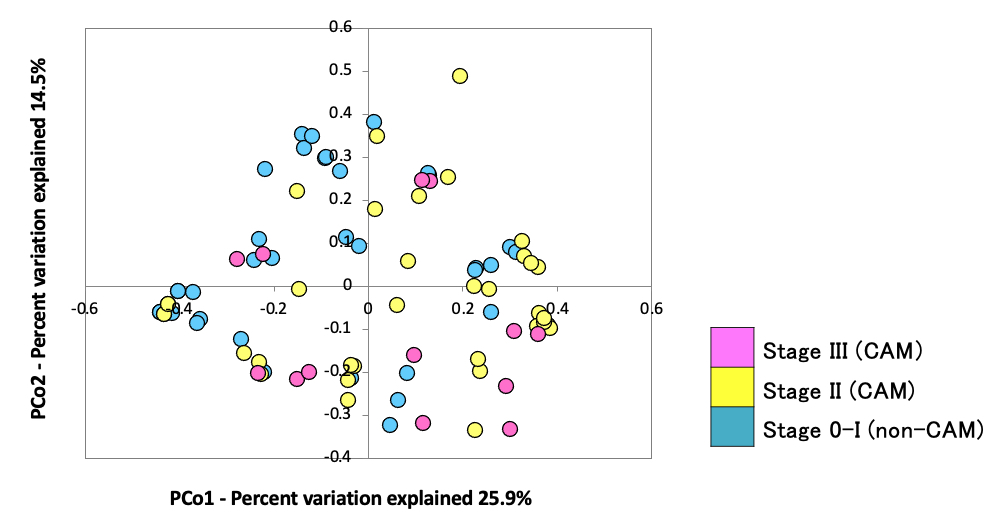


**Supplementary Figure S1. Principal coordinate analysis based on unweighted UniFrac distances.** Amplicons of 16S rDNA were sequenced using 27Fmod and 338R primers. The multidimensional composition of each group was determined based on the matrix data for the unweighted UniFrac distance. Each group appeared scattered without any clusters. PCo1, principal coordinate 1; PCo2, principal coordinate 2; CAM, chorioamnionitis.

**
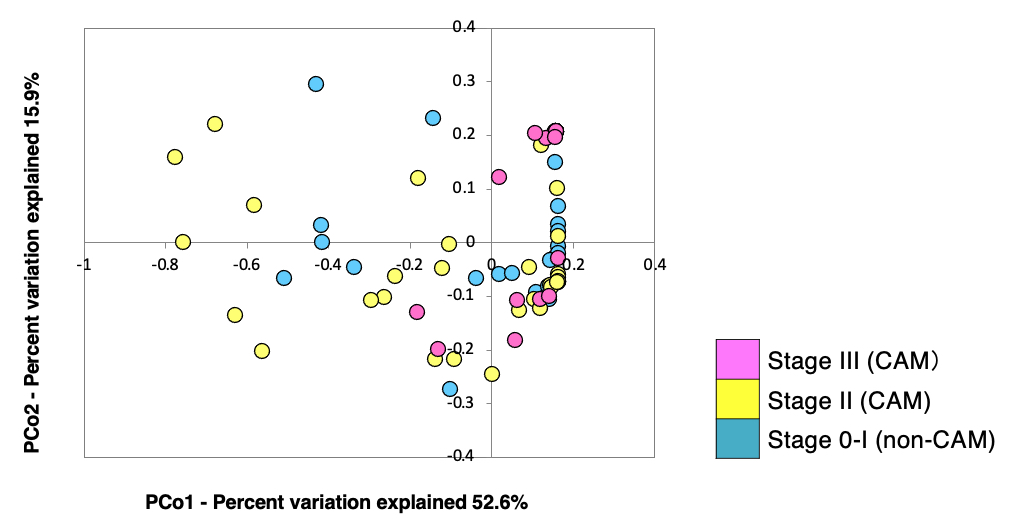
**

**Supplementary Figure S2. Principal coordinate analysis based on weighted UniFrac distances.** Amplicons of 16S rDNA were sequenced using 27Fmod and 338R primers. The multidimensional composition of each group was determined based on the matrix data for the weighted UniFrac distance. Each group appeared scattered without any clusters. PCo1, principal coordinate 1; PCo2, principal coordinate 2; CAM, chorioamnionitis.

**
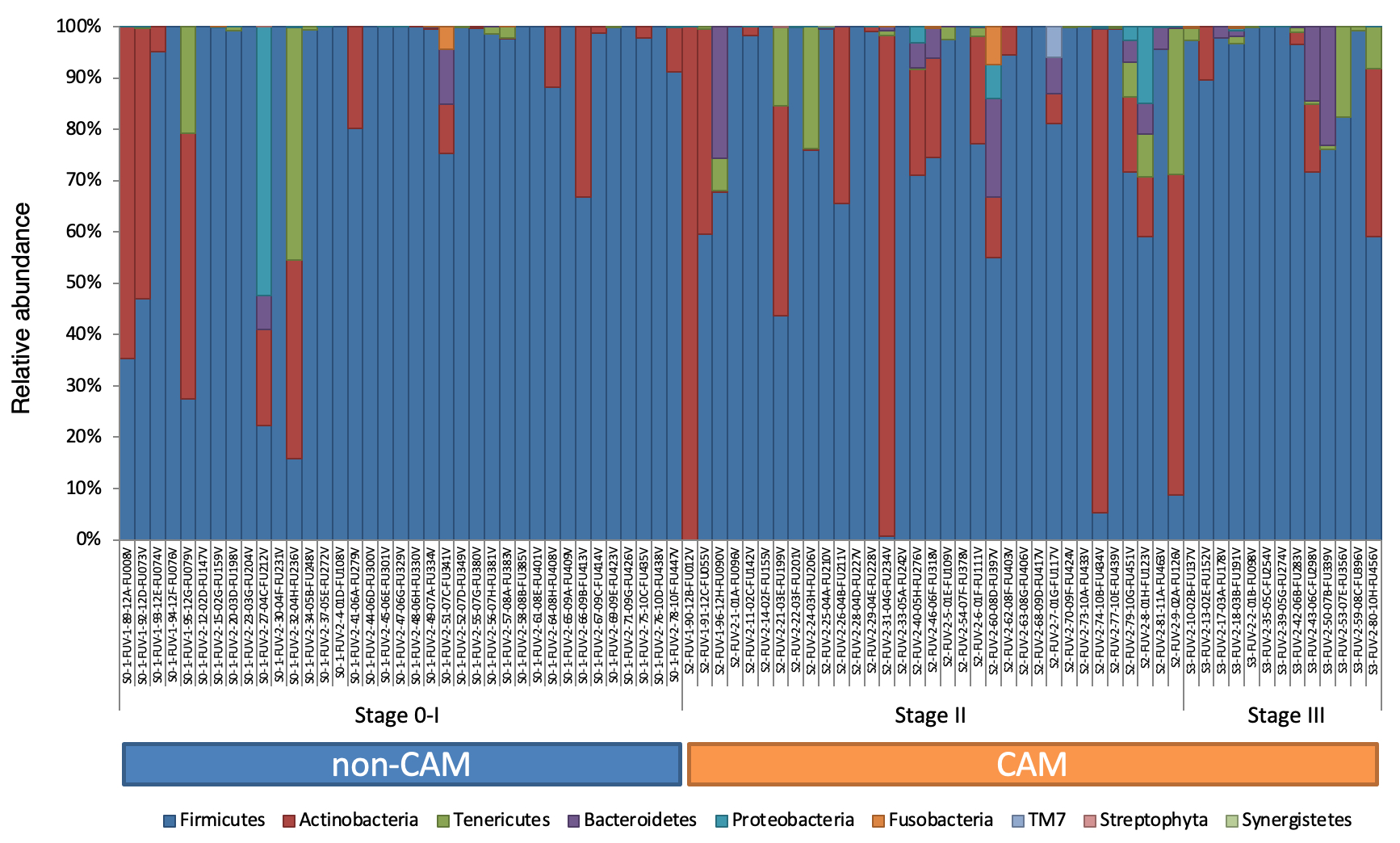
**

**Supplementary Figure S3. Relative abundances of different bacterial phyla in each sample.** Sequences were clustered into operational taxonomic units with a 70% identity threshold, and taxonomic assignments were performed by a similarity search against the standard database. Any significant differences based on chorioamnionitis (CAM) and staging (Blanc’s classification) were not shown in the compositions at the phylum level.

**
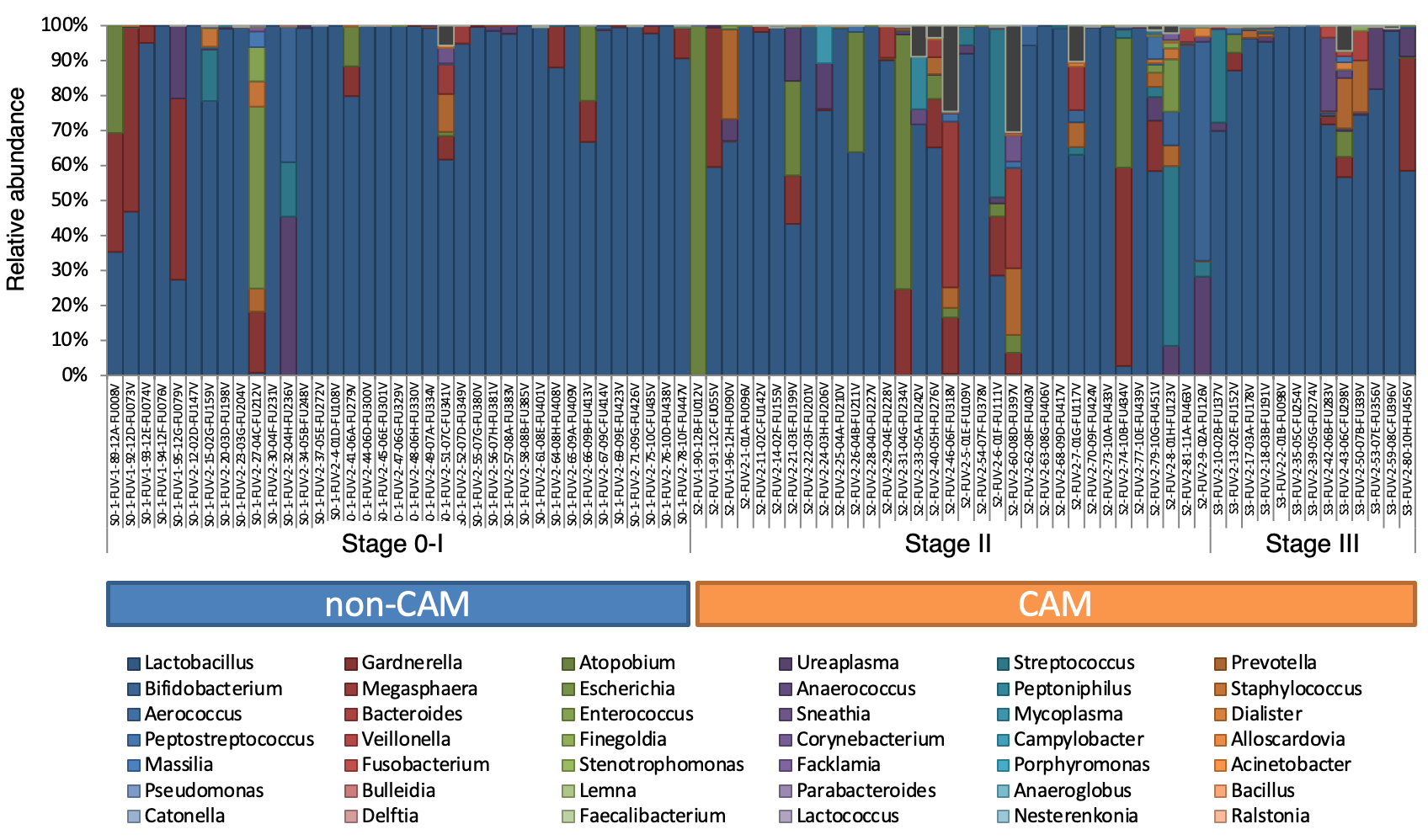
**

**Supplementary Figure S4. Relative abundances of different bacterial genera in each sample.** Sequences were clustered into operational taxonomic units with a 94% identity threshold, and taxonomic assignments were performed by a similarity search against the standard database. No significant differences based on chorioamnionitis (CAM) and staging (Blanc’s classification) were observed in the compositions at the genus level.

**
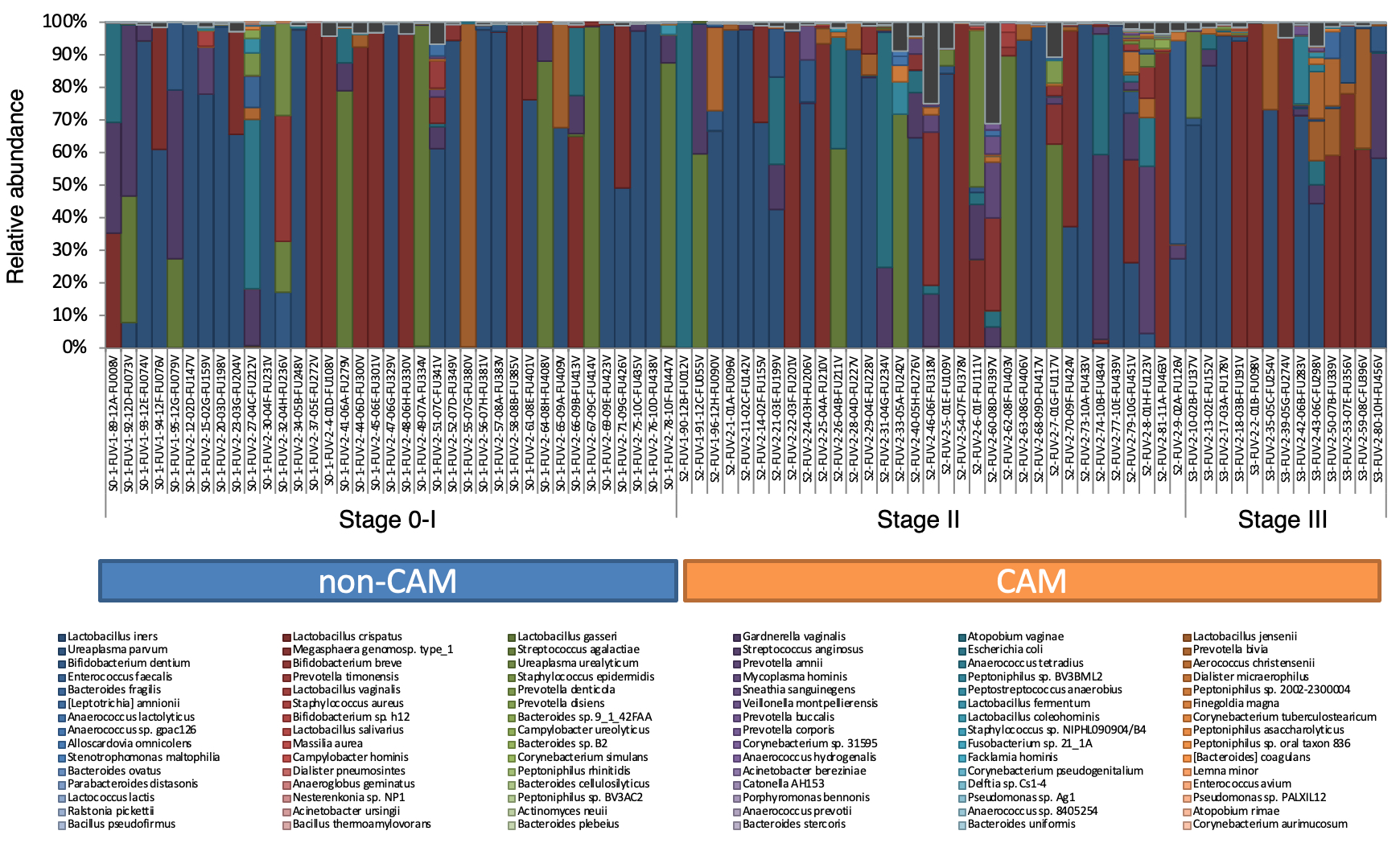
**

**Supplementary Figure S5. Relative abundances of different bacterial species in each sample.** Sequences were clustered into operational taxonomic units with a 97% identity threshold, and taxonomic assignments were performed by a similarity search against the standard database. No significant differences based on chorioamnionitis (CAM) and staging (Blanc’s classification) were observed in the compositions at the species level.

**
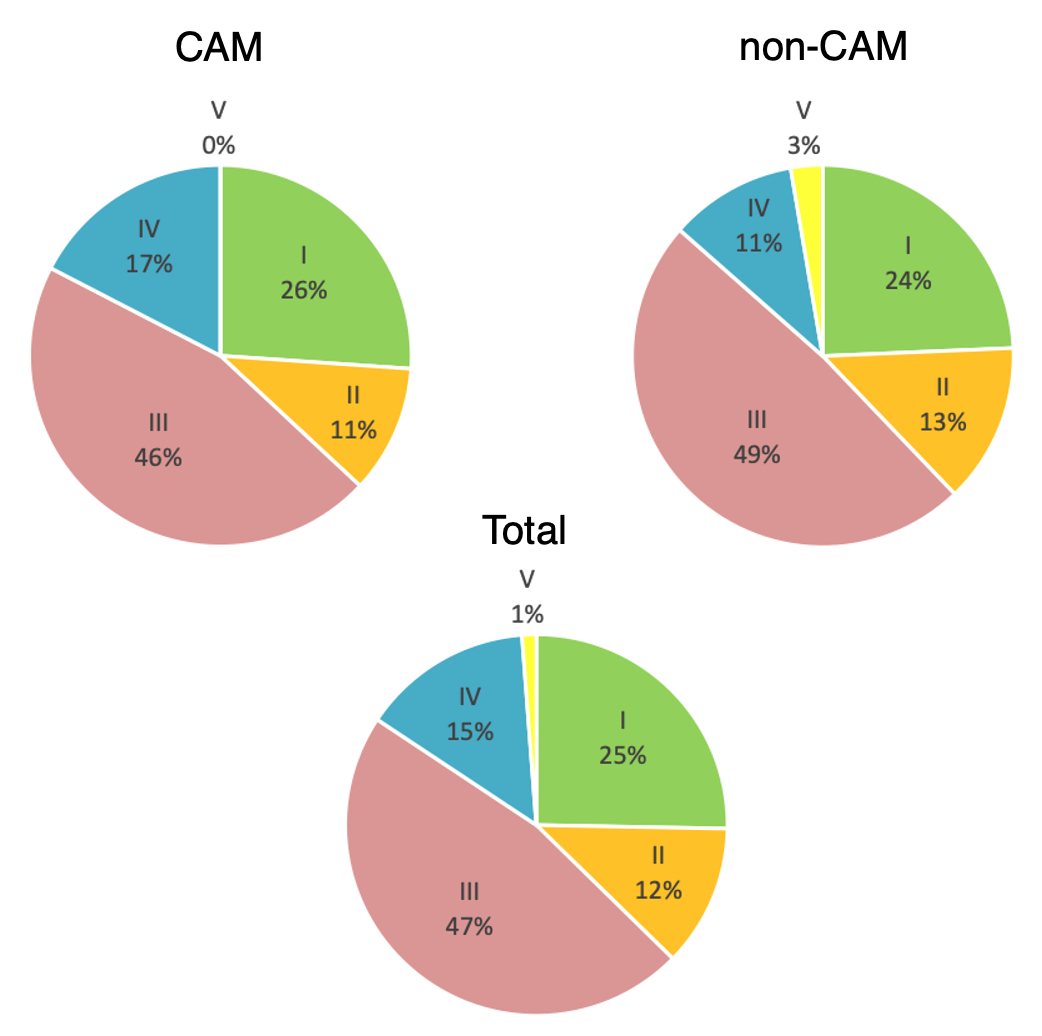
**

**Supplementary Figure S6. Distribution of vaginal community state type (CST).** Five types of vaginal bacterial CSTs were classified according to previous reports. Four of these were dominated by *Lactobacillus* spp., including *L. crispatus* (CST I), *L. gasseri* (CST II), *L. iners* (CST III), and *L. jensenii* (CST V). CST IV was characterized by small relative abundances of *Lactobacillus* spp. and increased relative abundances of anaerobic bacteria. No significant differences based on chorioamnionitis (CAM) and staging (Blanc’s classification) were observed in the frequencies of CSTs.

**Supplementary Tables**

**Supplementary Table S1. Group definitions**

| **Group name** | **Sample number** | **Blanc’s classification (symbol)** | **Sample number** |
| --- | --- | --- | --- |
| CAM | 46 | Stage III (S3) | 13 |
|  |  | Stage II (S2) | 33 |
| non-CAM | 37 | Stage I (S1) | 19 |
|  |  | No neutrophil infiltration (S0) | 18 |

**Supplementary Table S2. Clinical characteristics and patient outcomes in each group**

|  | **CAM** | **non-CAM** | ***P*-value** |
| --- | --- | --- | --- |
|  | **(n=46)** | **(n=37)** |  |
| **Maternal data** |  |  |  |
| Gestational age at sampling (weeks)^†^ | 30.7 (25.1–33.8) | 32.1 (29.4–34.0) | 0.118 |
| Age^†^ | 30.5 (27.0–35.8) | 33.0 (29.0–35.0) | 0.140 |
| Gravidity^†^ | 2.0 (1.0–3.0) | 2.0 (1.0–3.0) | 0.806 |
| Parity^†^ | 0.5 (0.0–1.0) | 0.0 (0.0–1.0) | 0.845 |
| Multiparity^§^ | 23 (50%) | 18 (49%) | 1.000 |
| History of smoking^§^ | 4 (9%) | 1 (3%) | 0.375 |
| After Shirodkar cerclage^§^ | 3 (7%) | 0 (0%) | 0.500 |
| History of arterial abortion^§^ | 14 (30%) | 6 (16%) | 0.038 |
| History of miscarriage or preterm birth^§^ | 9 (20%) | 15 (41%) | 0.051 |
| History of cesarean section^§^ | 2 (4%) | 6 (16%) | 0.131 |
| Congenital anomalies of the uterus^§^ | 0 (0%) | 1 (3%) | 0.446 |
| Twins^§^ | 0 (0%) | 1 (3%) | 0.446 |
| Preeclampsia or superimposed preeclampsia^§^ | 6 (13%) | 6 (16%) | 0.759 |
| Hydramnios with preterm labor^§^ | 0 (0%) | 0 (0%) | ND |
| Preterm premature rupture of membranes after sampling^§^ | 1 (2%) | 3 (8%) | 0.319 |
| Antibiotics before sampling^§^ | 1 (2%) | 0 (0%) | 1.000 |
| Antenatal corticosteroid before sampling^§^ | 3 (7%) | 1 (3%) | 0.625 |
| Tocolytic agent after sampling^§^ | 40 (87%) | 31 (84%) | 0.759 |
| Arterial abortion^§^ | 0 (0%) | 1 (3%) | 0.446 |
| IUFD^§^ | 1 (2%) | 0 (0%) | 1.000 |
| Threatened rupture of the uterus (repeat CS + preterm labor)^§^ | 1 (2%) | 3 (8%) | 0.319 |
| Cesarean section^§^ | 17 (37%) | 25 (68%) | 0.008 |
| Cervical length at sampling (mm)^‡^ | 14.5 (0.0–21.0; 34) | 25.0 (14.0–34.3; 32) | 0.002 |
| Body temperature at sampling (°C)^†^ | 37.0 (36.7–37.1) | 37.0 (36.7–37.3) | 0.800 |
| Heart rate at sampling (/min)^†^ | 92.5 (82.0–101.5) | 85.0 (74.0–96.0) | 0.019 |
| WBC count in maternal peripheral blood at sampling (cells/µL)^†^ | 11,200 (8,700–14,100) | 9,600 (7,700–11,400) | 0.082 |
| CRP in maternal peripheral blood at sampling (mg/dL)^†^ | 0.5 (0.1–1.6) | 0.4 (0.1–0.9) | 0.292 |
| Clinical chorioamnionitis at sampling^§^ | 0 (0%) | 0 (0%) | ND |
| Staging of chorioamnionitis (Blanc’s classification)^†^ | 2.0 (2.0–3.0) | 1.0 (0.0–1.0) | <0.001 |
| Chorioamnionitis (Blanc’s stage II–III)^§^ | 46 (100%) | 0 (0%) | <0.001 |
| Funisitis of umbilical cord^§^ | 8/45 (18%) | 0 (0%) | 0.007 |
| Pregnancy extension period from admission to birth (days)^†^ | 4.0 (1.0–19.5) | 13.0 (1.0–35.0) | 0.309 |
| Major complications after birth^†^ | 1 (2%) | 0 (0%) | 1.000 |
| Days of hospital stay after birth (days)^†^ | 4.0 (4.0–5.0) | 5.0 (4.0–5.0) | 0.009 |
| **Neonatal data** |  |  |  |
| Gestational age at birth (weeks)^†^ | 33.9 (27.0–36.0) | 35.6 (33.3–37.0) | 0.036 |
| Extremely preterm birth (GA <28 weeks)^§^ | 12 (26%) | 2 (5%) | 0.017 |
| Very preterm birth (GA <32 weeks)^§^ | 18 (39%) | 7 (19%) | 0.056 |
| Preterm birth (GA <36 weeks)^§^ | 33 (72%) | 22 (59%) | 0.254 |
| Preterm birth (GA <37 weeks)^§^ | 39 (85%) | 27 (73%) | 0.274 |
| Neonatal body weight at birth (g)^‡^ | 1,814 (904–2,301; 47) | 2,146 (1,460–2,498; 36) | 0.035 |
| Severe anomaly/chromosomal disease^§^ | 0 (0%) | 3 (8%) | 0.085 |
| SGA^¶^ | 5/42 (12%) | 8/35 (23%) | 0.234 |
| Male^¶^ | 20/42 (48%) | 20/35 (57%) | 0.494 |
| Apgar score 1 min^‡^ | 8.0 (4.3–8.0; 42) | 7.0 (4.5–8.0; 35) | 0.673 |
| Apgar score 5 min^‡^ | 9.0 (8.0–9.0; 42) | 9.0 (8.0–9.0; 35) | 0.880 |
| Umbilical arterial pH at birth^‡^ | 7.33 (7.30–7.40; 40) | 7.32 (7.24–7.34; 33) | 0.071 |
| WBC count after birth (cells/µL)^‡^ | 13,050 (9,125–17,550; 36) | 11,850 (9,350–13,675; 26) | 0.336 |
| CRP after birth (mg/dL)^‡^ | 0.0 (0.0–0.0; 36) | 0.0 (0.0–0.0; 26) | 0.054 |
| IgM after birth (mg/dL)^‡^ | 9.0 (7.0–12.3; 36) | 7.0 (6.0–9.0; 25) | 0.011 |
| Admission in NICU^¶^ | 36/42 (86%) | 28/35 (80%) | 0.553 |
| Antibiotics for newborn^¶^ | 9/42 (21%) | 5/35 (14%) | 0.556 |
| Sepsis^¶^ | 1/42 (2%) | 0/35 (0%) | 1.000 |
| Developmental disorders at 1.5 years old^¶^ | 7/37 (19%) | 6/34 (18%) | 1.000 |
| Developmental disorders at 3 years old^¶^ | 7/36 (19%) | 6/34 (18%) | 1.000 |
| Cerebral palsy^¶^ | 1/42 (2%) | 2/35 (6%) | 0.588 |
| Chronic lung disease^¶^ | 8/42 (19%) | 1/35 (3%) | 0.035 |
| EEG abnormality^¶^ | 4/42 (10%) | 0/35 (0%) | 0.121 |
| Brain MRI abnormality^¶^ | 3/42 (7%) | 0/35 (0%) | 0.246 |

Data are presented as ^†^medians (interquartile ranges), ^‡^medians (interquartile ranges; n), ^§^n (%), or ^¶^n/N (%).

IUFD, intrauterine fetal death; WBC, white blood cell; CRP, C-reactive protein; CS, cesarean section; GA, gestational age; SGA, small for gestational age; IgM, immunoglobulin M; NICU, neonatal intensive care unit; EEG, electroencephalogram; MRI, magnetic resonance imaging; CAM, chorioamnionitis.

**Supplementary Table S3. Statistical analysis of the UniFrac distance in each group**

| **Category** | **Weighted UniFrac** | | **Unweighted UniFrac** | |
| --- | --- | --- | --- | --- |
|  | **R^2^** | ***P*-value** | **R^2^** | ***P*-value** |
| S0–1 vs S2–3 | 0.023 | 0.533 | 0.052 | <0.001 |
| S0–1 vs S2 | 0.039 | 0.155 | 0.053 | 0.004 |
| S0–1 vs S3 | 0.015 | 0.155 | 0.053 | 0.017 |
| S2 vs S3 | 0.042 | 0.155 | 0.022 | 0.462 |

**Supplementary Table S5. Extraction of bacterial species related to CAM using the random forest algorithm**

| **Name of bacterial species** | **Mean decrease accuracy** | **Predominant group** |
| --- | --- | --- |
| *Finegoldia magna* | 6.91 | CAM |
| *Streptococcus anginosus* | 5.58 | CAM |
| *Aerococcus christensenii* | 4.85 | CAM |
| *Pseudomonas* sp. Ag1 | 4.12 | CAM |
| *Peptoniphilus* sp. BV3BML2 | 3.52 | CAM |
| *Lactobacillus jensenii* | 3.13 | CAM |
| *Ureaplasma parvum* | 2.74 | CAM |
| *Prevotella disiens* | 2.55 | CAM |
| *Lactobacillus vaginalis* | 2.50 | CAM |
| *Prevotella buccalis* | 2.31 | CAM |
| *Dialister micraerophilus* | 2.12 | CAM |
| *Atopobium vaginae* | 2.01 | CAM |
| *Prevotella bivia* | 1.82 | CAM |
| *Peptoniphilus* sp. 2002.2300004 | 1.59 | CAM |
| *Prevotella amnii* | 1.58 | CAM |
| *Anaerococcus lactolyticus* | 1.41 | CAM |
| *Streptococcus agalactiae* | 1.27 | CAM |
| *Anaerococcus tetradius* | 1.03 | CAM |
| *Lactobacillus gasseri* | 2.21 | non-CAM |
| *Enterococcus avium* | 1.42 | non-CAM |

CAM, chorioamnionitis.

**Supplementary Table S6. Comparison of predictive diagnostic accuracy**

|  | **AUC (95%CI)** | **Cutoff value** | **Sensitivity** | **Specificity** | **Youden index** |
| --- | --- | --- | --- | --- | --- |
| PCAM score | 0.849 (0.765–0.934) | 1.5 | 0.714 | 0.824 | 0.538 |
| Chao1 | 0.760 (0.650–0.870) | 9.8 | 0.571 | 0.882 | 0.453 |
| Shannon’s index | 0.683 (0.562–0.805) | 0.26 | 0.762 | 0.588 | 0.350 |
| Cervical length at sampling (mm) | 0.702 (0.582–0.823) | 21.5 | 0.786 | 0.588 | 0.374 |
| Body temperature at sampling (°C) | 0.506 (0.373–0.639) | 36.5 | 0.881 | 0.206 | 0.087 |
| Heart rate at sampling (/min) | 0.648 (0.526–0.771) | 96.5 | 0.476 | 0.765 | 0.241 |
| WBC count in maternal peripheral blood at sampling (cells/µL) | 0.625 (0.498–0.752) | 12,150 | 0.524 | 0.794 | 0.318 |
| CRP in maternal peripheral blood at sampling (mg/dL) | 0.546 (0.415–0.676) | 0.75 | 0.452 | 0.735 | 0.187 |

PCAM, predictive chorioamnionitis; WBC, white blood cell; CRP, C-reactive protein; AUC, area under the curve; CI, confidence interval.

**Supplementary Table S7. Clinical characteristics and outcomes of patients in each group in the sub-analysis**

|  | **CAM (n=30)** | **non-CAM (n=17)** | ***P*-value** |
| --- | --- | --- | --- |
| **Maternal data** |  |  |  |
| Gestational age at sampling (weeks)^†^ | 29.9 (25.6–33.4) | 30.7 (29.4–33.3) | 0.203 |
| Age^†^ | 29.5 (27.0–34.8) | 31.0 (28.0–35.0) | 0.346 |
| Gravidity^†^ | 2.0 (1.0–2.8) | 2.0 (1.0–3.0) | 0.858 |
| Parity^†^ | 1.0 (0.0–1.0) | 0.0 (0.0–1.0) | 0.593 |
| Multiparity^§^ | 16 (53%) | 7 (41%) | 0.547 |
| History of smoking^§^ | 2 (7%) | 1 (6%) | ND |
| After Shirodkar cerclage^§^ | 0 (0%) | 0 (0%) | ND |
| History of arterial abortion^§^ | 9 (30%) | 4 (24%) | 0.380 |
| History of miscarriage or preterm birth^§^ | 3 (10%) | 8 (47%) | 0.009 |
| History of cesarean section^§^ | 0 (0%) | 3 (18%) | 0.042 |
| Congenital anomalies of the uterus^§^ | 0 (0%) | 0 (0%) | ND |
| Twins^§^ | 0 (0%) | 0 (0%) | ND |
| Preeclampsia or superimposed preeclampsia^§^ | 0 (0%) | 0 (0%) | ND |
| Hydramnios with preterm labor^§^ | 0 (0%) | 0 (0%) | ND |
| Preterm premature rupture of membranes after sampling^§^ | 1 (3%) | 2 (12%) | 0.544 |
| Antibiotics before sampling^§^ | 1 (3%) | 0 (0%) | 1.000 |
| Antenatal corticosteroid before sampling^§^ | 2 (7%) | 1 (6%) | 1.000 |
| Tocolytic agent after sampling^§^ | 30 (100%) | 15 (88%) | 0.126 |
| Arterial abortion^§^ | 0 (0%) | 0 (0%) | ND |
| IUFD^§^ | 0 (0%) | 0 (0%) | ND |
| Threatened rupture of the uterus (repeat CS + preterm labor)^§^ | 0 (0%) | 0 (0%) | ND |
| Cesarean section^§^ | 10 (33%) | 11 (65%) | 0.066 |
| Cervical length at sampling (mm)^†^ | 10.6 (0.0–19.0) | 27.0 (13.0–35.6) | 0.002 |
| Body temperature at sampling (°C)^†^ | 36.9 (36.6–37.1) | 36.9 (36.4–37.3) | 0.763 |
| Heart rate at sampling (/min)^†^ | 92.5 (82.3–100.0) | 82.0 (78.0–90.0) | 0.017 |
| WBC count in maternal peripheral blood at sampling (cells/µL)^†^ | 12,300 (9,000–14,100) | 10,200 (8,300–11,400) | 0.150 |
| CRP in maternal peripheral blood at sampling (mg/dL)^†^ | 0.3 (0.1–1.2) | 0.2 (0.1–0.5) | 0.572 |
| Clinical chorioamnionitis at sampling^§^ | 0 (0%) | 0 (0%) | ND |
| Staging of chorioamnionitis (Blanc’s classification)^†^ | 2.0 (2.0–3.0) | 0.0 (0.0–1.0) | <0.001 |
| Chorioamnionitis (Blanc’s stage II–III)^§^ | 30 (100%) | 0 (0%) | <0.001 |
| Funisitis of umbilical cord^§^ | 6 (20%) | 0 (0%) | 0.074 |
| Pregnancy extension period from admission to birth (days)^†^ | 4.5 (1.0–18.0) | 21.0 (14.0–46.0) | 0.003 |
| Major complications after birth^§^ | 1 (3%) | 0 (0%) | 1.000 |
| Days of hospital stay after birth (days)^†^ | 4.0 (4.0–5.0) | 5.0 (4.0–5.0) | 0.023 |
| **Neonatal data** |  |  |  |
| Gestational age at birth (weeks)^†^ | 32.9 (26.8–35.3) | 35.9 (34.3–37.0) | 0.003 |
| Extremely preterm birth (GA <28 weeks)^§^ | 8 (27%) | 0 (0%) | 0.038 |
| Very preterm birth (GA <32 weeks)^§^ | 14 (47%) | 2 (12%) | 0.024 |
| Preterm birth (GA <36 weeks)^§^ | 25 (83%) | 9 (53%) | 0.041 |
| Preterm birth (GA <37 weeks)^§^ | 28 (93%) | 12 (70%) | 0.081 |
| Neonatal body weight at birth (g)^†^ | 1,780 (904–2,240) | 2,147 (1,877–2,605) | 0.010 |
| Severe anomaly/chromosomal disease^§^ | 0 (0%) | 0 (0%) | ND |
| SGA^¶^ | 2/28 (7%) | 2/17 (12%) | 0.626 |
| Male^¶^ | 14/28 (50%) | 11/17 (65%) | 0.372 |
| Apgar score 1 min^‡^ | 7.0 (4.8–8.0; 17) | 8.0 (7.0–8.0; 28) | 0.494 |
| Apgar score 5 min^‡^ | 8.0 (7.0–9.0; 17) | 9.0 (8.0–9.0; 28) | 0.293 |
| Umbilical arterial pH at birth^‡^ | 7.33 (7.30–7.39; 17) | 7.34 (7.26–7.35; 26) | 0.472 |
| WBC count after birth (cells/µL)^‡^ | 13,700 (9,300–17,250; 13) | 11,000 (9,200–13,800; 25) | 0.296 |
| CRP after birth (mg/dL)^‡^ | 0.0 (0.0–0.0; 13) | 0.0 (0.0–0.0; 25) | 0.307 |
| IgM after birth (mg/dL)^‡^ | 9.0 (8.0–13.0; 13) | 8.0 (6.0–9.0; 25) | 0.154 |
| Admission in NICU^¶^ | 25/28 (89%) | 14/17 (82%) | 0.658 |
| Antibiotics for newborn^¶^ | 4/28 (14%) | 2/17 (12%) | 1.000 |
| Sepsis^¶^ | 1/28 (4%) | 0/17 (0%) | 1.000 |
| Developmental disorders at 1.5 years old^¶^ | 4/25 (16%) | 1/17 (6%) | 0.632 |
| Developmental disorders at 3 years old^¶^ | 4/24 (17%) | 2/17 (12%) | 1.000 |
| Cerebral palsy^¶^ | 0/28 (0%) | 1/17 (6%) | 0.378 |
| Chronic lung disease^¶^ | 6/28 (21%) | 0/17 (0%) | 0.069 |
| EEG abnormality^¶^ | 3/28 (11%) | 0/17 (0%) | 0.279 |
| Brain MRI abnormality^¶^ | 2/28 (7%) | 0/17 (0%) | 0.519 |

Data are presented as ^†^medians (interquartile ranges), ^‡^medians (interquartile ranges; n), ^§^n (%), or ^¶^n/N (%).

IUFD, intrauterine fetal death; CS, cesarean section; WBC, white blood cell; CRP, C-reactive protein; GA, gestational age; SGA, small for gestational age; IgM, immunoglobulin M; NICU, neonatal intensive care unit; EEG, electroencephalogram; MRI, magnetic resonance imaging; CAM, chorioamnionitis.

**Supplementary Table S8. Comparison of predictive diagnostic accuracy in the sub-analysis**

|  | **AUC (95%CI)** | **Cutoff value** | **Sensitivity** | **Specificity** | **Youden index** |
| --- | --- | --- | --- | --- | --- |
| PCAM score | 0.843 (0.734–0.952) | 1.5 | 0.667 | 0.882 | 0.549 |
| Chao1 | 0.793 (0.657–0.929) | 4.5 | 0.833 | 0.647 | 0.480 |
| Shannon’s index | 0.680 (0.514–0.846) | 0.22 | 0.767 | 0.588 | 0.355 |
| Cervical length at sampling (mm) | 0.764 (0.620–0.908) | 19.5 | 0.767 | 0.706 | 0.473 |
| Heart rate at sampling (/min) | 0.709 (0.560–0.858) | 96.5 | 0.467 | 0.941 | 0.408 |
| WBC count in maternal peripheral blood at sampling (cells/µL) | 0.628 (0.459–0.798) | 11,500 | 0.567 | 0.765 | 0.332 |
| CRP in maternal peripheral blood at sampling (mg/dL) | 0.551 (0.380–0.722) | 0.70 | 0.400 | 0.824 | 0.224 |

PCAM, predictive chorioamnionitis; WBC, white blood cell; CRP, C-reactive protein; AUC, area under the curve.

**Supplementary Table S9. Comparison of perinatal outcomes between the PCAM and non-PCAM groups in the sub-analysis**

|  | **PCAM (n=22)** | **non-PCAM (n=25)** | ***P*-value** |
| --- | --- | --- | --- |
| **Maternal data** |  |  |  |
| Gestational age at sampling (weeks)^†^ | 30.7 (27.3–33.9) | 30.4 (27.6–32.9) | 0.763 |
| Age^†^ | 29.0 (27.0–31.0) | 32.0 (28.0–36.0) | 0.070 |
| Gravidity^†^ | 2.0 (1.0–2.0) | 2.0 (1.0–3.0) | 0.887 |
| Parity^†^ | 0.5 (0.0–1.0) | 0.0 (0.0–1.0) | 0.894 |
| Multiparity^§^ | 11 (50%) | 12 (48%) | 1.000 |
| History of smoking^§^ | 1 (5%) | 2 (8%) | 1.000 |
| After Shirodkar cerclage^§^ | 0 (0%) | 0 (0%) | ND |
| History of arterial abortion^§^ | 6 (27%) | 7 (28%) | ND |
| History of miscarriage or preterm birth^§^ | 2 (9%) | 9 (36%) | ND |
| History of cesarean section^§^ | 0 (0%) | 3 (12%) | 0.237 |
| Congenital anomalies of the uterus^§^ | 0 (0%) | 0 (0%) | ND |
| Twins^§^ | 0 (0%) | 0 (0%) | ND |
| Preeclampsia or superimposed preeclampsia^§^ | 0 (0%) | 0 (0%) | ND |
| Hydramnios with preterm labor^§^ | 0 (0%) | 0 (0%) | ND |
| Preterm premature rupture of membranes after sampling^§^ | 1 (5%) | 2 (8%) | 1.000 |
| Antibiotics before sampling^§^ | 0 (0%) | 1 (4%) | 1.000 |
| Antenatal corticosteroid before sampling^§^ | 0 (0%) | 3 (12%) | 0.237 |
| Tocolytic agent after sampling^§^ | 22 (100%) | 23 (92%) | 0.491 |
| Arterial abortion^§^ | 0 (0%) | 0 (0%) | ND |
| IUFD^§^ | 0 (0%) | 0 (0%) | ND |
| Threatened rupture of the uterus (repeat CS + preterm labor)^§^ | 0 (0%) | 0 (0%) | ND |
| Cesarean section^§^ | 8 (36%) | 13 (52%) | 0.381 |
| Cervical length at sampling (mm)^†^ | 10.6 (0.0–19.0) | 20.0 (8.7–30.0) | 0.065 |
| Body temperature at sampling (°C)^†^ | 36.9 (36.6–37.1) | 36.8 (36.4–37.2) | 0.878 |
| Heart rate at sampling (/min)^†^ | 92.5 (85.0–100.0) | 83.0 (79.0–92.0) | 0.022 |
| WBC count in maternal peripheral blood at sampling (cells/µL)^†^ | 12,550 (8,875–14,400) | 10,500 (8,400–12,600) | 0.135 |
| CRP in maternal peripheral blood at sampling (mg/dL)^†^ | 0.4 (0.1–1.2) | 0.2 (0.1–0.8) | 0.475 |
| Clinical chorioamnionitis at sampling^§^ | 0 (0%) | 0 (0%) | ND |
| Staging of chorioamnionitis (Blanc’s classification)^†^ | 2.0 (2.0–3.0) | 1.0 (0.0–2.0) | 0.002 |
| Chorioamnionitis (Blanc’s stage II–III)^§^ | 20 (91%) | 10 (40%) | 0.001 |
| Funisitis of umbilical cord^§^ | 4 (18%) | 2 (8%) | 0.398 |
| Pregnancy extension period from admission to birth (days)^†^ | 4.0 (1.0–18.0) | 17.0 (5.0–46.0) | 0.022 |
| Major complications after birth^§^ | 0 (0%) | 1 (4%) | 1.000 |
| Days of hospital stay after birth (days)^†^ | 4.0 (4.0–5.0) | 5.0 (4.0–5.0) | 0.122 |
| **Neonatal data** |  |  |  |
| Gestational age at birth (weeks)^†^ | 33.4 (30.9–35.4) | 35.3 (31.6–36.9) | 0.087 |
| Extremely preterm birth (GA <28 weeks)^§^ | 4 (18%) | 4 (16%) | 1.000 |
| Very preterm birth (GA <32 weeks)^§^ | 9 (41%) | 7 (28%) | 0.376 |
| Preterm birth (GA <36 weeks)^§^ | 19 (86%) | 15 (60%) | 0.056 |
| Preterm birth (GA <37 weeks)^§^ | 21 (95%) | 19 (76%) | 0.102 |
| Neonatal body weight at birth (g)^†^ | 1,888 (1,137–2,240) | 2,103 (1,215–2,412) | 0.153 |
| Severe anomaly/chromosomal disease^§^ | 0 (0%) | 0 (0%) | ND |
| SGA^¶^ | 1/21 (5%) | 3/24 (13%) | 0.611 |
| Male^¶^ | 12/21 (57%) | 13/24 (54%) | 1.000 |
| Apgar score 1 min^‡^ | 7.0 (5.0–8.0; 24) | 8.0 (6.0–8.0; 21) | 0.285 |
| Apgar score 5 min^‡^ | 8.0 (7.0–9.0; 24) | 9.0 (8.0–9.0; 21) | 0.087 |
| Umbilical arterial pH at birth^‡^ | 7.34 (7.30–7.39; 23) | 7.33 (7.26–7.36; 20) | 0.534 |
| WBC count after birth (cells/µL)^‡^ | 15,500 (9,250–17,150; 19) | 10,600 (9,200–13,450; 19) | 0.136 |
| CRP after birth (mg/dL)^‡^ | 0.0 (0.0–0.0; 19) | 0.0 (0.0–0.0; 19) | 0.743 |
| IgM after birth (mg/dL)^‡^ | 9.0 (7.5–13.5; 19) | 8.0 (7.0–9.5; 19) | 0.347 |
| Admission in NICU^¶^ | 19/21 (90%) | 20/24 (83%) | 0.670 |
| Antibiotics for newborn^¶^ | 3/21 (14%) | 3/24 (13%) | 1.000 |
| Sepsis^¶^ | 0/21 (0%) | 1/24 (4%) | 1.000 |
| Developmental disorders at 1.5 years old^¶^ | 3/19 (16%) | 2/23 (9%) | 0.644 |
| Developmental disorders at 3 years old^¶^ | 5/18 (28%) | 1/23 (4%) | 0.070 |
| Cerebral palsy^¶^ | 1/21 (5%) | 0/24 (0%) | 0.467 |
| Chronic lung disease^¶^ | 3/21 (14%) | 3/24 (13%) | 1.000 |
| EEG abnormality^¶^ | 2/21 (10%) | 1/24 (4%) | 0.592 |
| Brain MRI abnormality^¶^ | 1/21 (5%) | 1/24 (4%) | 1.000 |

Data are presented as ^†^medians (interquartile ranges), ^‡^medians (interquartile ranges; n), ^§^n (%), or ^¶^n/N (%).

PCAM, predictive chorioamnionitis; IUFD, intrauterine fetal death; CS, cesarean section; WBC, white blood cell; CRP, C-reactive protein; GA, gestational age; SGA, small for gestational age; IgM, immunoglobulin M; NICU, neonatal intensive care unit; EEG, electroencephalogram; MRI, magnetic resonance imaging.

**Supplementary Table S10. The crude and multivariable odds ratios for predictive chorioamnionitis**

|  | **PCAM (n=22)** | **non-PCAM (n=25)** | **Crude OR (95% CI)** | **Crude *P*-value** | **Multivariable OR (95% CI)** | **Multivariable *P-*value** |
| --- | --- | --- | --- | --- | --- | --- |
| Developmental disorders at 3 years old | 5/18 (28%) | 1/23 (4%) | 8.46 (1.19–171.69) | 0.031 | 10.93 (1.37–288.33) | 0.022 |
| SGA | 1/21 (5%) | 3/24 (13%) | 0.35 (0.02–2.99) | 0.351 | 0.23 (0.01–2.62) | 0.253 |

PCAM, predictive chorioamnionitis; SGA, small for gestational age; OR, odds ratio; CI, confidence interval.

**Supplementary Table S11. Summary of sequence reads**

| **Group symbol** | **Sample number** | **Sample ID** | **Number of reads in input for quality filtering** | **Reads removed** | | |
| --- | --- | --- | --- | --- | --- | --- |
|  |  |  |  | **Reads lacking primer sequences** | **Reads with average quality <25** | **Possible chimeric reads** |
| S0-1 | 1 | S0-1-FUV-1-89-12A-FU008V | 10,000 | 54 | 30 | 41 |
| S0-1 | 2 | S0-1-FUV-1-92-12D-FU073V | 10,000 | 48 | 40 | 35 |
| S0-1 | 3 | S0-1-FUV-1-93-12E-FU074V | 8,672 | 40 | 13 | 26 |
| S0-1 | 4 | S0-1-FUV-1-94-12F-FU076V | 10,000 | 37 | 11 | 40 |
| S0-1 | 5 | S0-1-FUV-1-95-12G-FU079V | 10,000 | 33 | 46 | 36 |
| S0-1 | 6 | S0-1-FUV-2-12-02D-FU147V | 10,000 | 39 | 5 | 13 |
| S0-1 | 7 | S0-1-FUV-2-15-02G-FU159V | 4,409 | 19 | 5 | 21 |
| S0-1 | 8 | S0-1-FUV-2-20-03D-FU198V | 8,467 | 42 | 18 | 10 |
| S0-1 | 9 | S0-1-FUV-2-23-03G-FU204V | 4,832 | 19 | 8 | 70 |
| S0-1 | 10 | S0-1-FUV-2-27-04C-FU212V | 10,000 | 115 | 30 | 47 |
| S0-1 | 11 | S0-1-FUV-2-30-04F-FU231V | 9,028 | 46 | 12 | 13 |
| S0-1 | 12 | S0-1-FUV-2-32-04H-FU236V | 10,000 | 98 | 51 | 185 |
| S0-1 | 13 | S0-1-FUV-2-34-05B-FU248V | 9,823 | 56 | 13 | 26 |
| S0-1 | 14 | S0-1-FUV-2-37-05E-FU272V | 4,807 | 22 | 2 | 3 |
| S0-1 | 15 | S0-1-FUV-2-4-01D-FU108V | 5,696 | 23 | 6 | 13 |
| S0-1 | 16 | S0-1-FUV-2-41-06A-FU279V | 4,913 | 26 | 2 | 38 |
| S0-1 | 17 | S0-1-FUV-2-44-06D-FU300V | 4,714 | 26 | 6 | 16 |
| S0-1 | 18 | S0-1-FUV-2-45-06E-FU301V | 5,118 | 17 | 3 | 4 |
| S0-1 | 19 | S0-1-FUV-2-47-06G-FU329V | 6,580 | 34 | 3 | 9 |
| S0-1 | 20 | S0-1-FUV-2-48-06H-FU330V | 4,686 | 14 | 1 | 7 |
| S0-1 | 21 | S0-1-FUV-2-49-07A-FU334V | 4,046 | 20 | 0 | 15 |
| S0-1 | 22 | S0-1-FUV-2-51-07C-FU341V | 2,987 | 13 | 4 | 107 |
| S0-1 | 23 | S0-1-FUV-2-52-07D-FU349V | 3,163 | 7 | 7 | 12 |
| S0-1 | 24 | S0-1-FUV-2-55-07G-FU380V | 4,722 | 20 | 5 | 9 |
| S0-1 | 25 | S0-1-FUV-2-56-07H-FU381V | 3,357 | 19 | 2 | 6 |
| S0-1 | 26 | S0-1-FUV-2-57-08A-FU383V | 3,572 | 18 | 1 | 10 |
| S0-1 | 27 | S0-1-FUV-2-58-08B-FU385V | 4,790 | 13 | 2 | 7 |
| S0-1 | 28 | S0-1-FUV-2-61-08E-FU401V | 5,253 | 31 | 4 | 97 |
| S0-1 | 29 | S0-1-FUV-2-64-08H-FU408V | 9,602 | 32 | 12 | 42 |
| S0-1 | 30 | S0-1-FUV-2-65-09A-FU409V | 10,000 | 51 | 13 | 123 |
| S0-1 | 31 | S0-1-FUV-2-66-09B-FU413V | 2,913 | 15 | 2 | 39 |
| S0-1 | 32 | S0-1-FUV-2-67-09C-FU414V | 5,442 | 32 | 1 | 18 |
| S0-1 | 33 | S0-1-FUV-2-69-09E-FU423V | 5,297 | 16 | 8 | 10 |
| S0-1 | 34 | S0-1-FUV-2-71-09G-FU426V | 7,359 | 36 | 3 | 114 |
| S0-1 | 35 | S0-1-FUV-2-75-10C-FU435V | 8,879 | 43 | 14 | 17 |
| S0-1 | 36 | S0-1-FUV-2-76-10D-FU438V | 9,230 | 34 | 16 | 10 |
| S0-1 | 37 | S0-1-FUV-2-78-10F-FU447V | 6,058 | 22 | 5 | 30 |
| S2 | 38 | S2-FUV-1-90-12B-FU012V | 10,000 | 25 | 17 | 56 |
| S2 | 39 | S2-FUV-1-91-12C-FU055V | 7,337 | 27 | 19 | 31 |
| S2 | 40 | S2-FUV-1-96-12H-FU090V | 10,000 | 33 | 21 | 87 |
| S2 | 41 | S2-FUV-2-1-01A-FU096V | 8,490 | 36 | 10 | 38 |
| S2 | 42 | S2-FUV-2-11-02C-FU142V | 10,000 | 67 | 11 | 59 |
| S2 | 43 | S2-FUV-2-14-02F-FU155V | 10,000 | 46 | 12 | 148 |
| S2 | 44 | S2-FUV-2-21-03E-FU199V | 2,322 | 13 | 7 | 31 |
| S2 | 45 | S2-FUV-2-22-03F-FU201V | 10,000 | 87 | 5 | 75 |
| S2 | 46 | S2-FUV-2-24-03H-FU206V | 10,000 | 38 | 15 | 35 |
| S2 | 47 | S2-FUV-2-25-04A-FU210V | 10,000 | 46 | 7 | 29 |
| S2 | 48 | S2-FUV-2-26-04B-FU211V | 5,944 | 28 | 4 | 37 |
| S2 | 49 | S2-FUV-2-28-04D-FU227V | 10,000 | 41 | 11 | 78 |
| S2 | 50 | S2-FUV-2-29-04E-FU228V | 8,109 | 42 | 7 | 73 |
| S2 | 51 | S2-FUV-2-31-04G-FU234V | 2,170 | 14 | 8 | 27 |
| S2 | 52 | S2-FUV-2-33-05A-FU242V | 10,000 | 49 | 3 | 70 |
| S2 | 53 | S2-FUV-2-40-05H-FU276V | 4,513 | 23 | 10 | 23 |
| S2 | 54 | S2-FUV-2-46-06F-FU318V | 1,754 | 9 | 8 | 22 |
| S2 | 55 | S2-FUV-2-5-01E-FU109V | 6,465 | 15 | 20 | 68 |
| S2 | 56 | S2-FUV-2-54-07F-FU378V | 3,643 | 9 | 5 | 2 |
| S2 | 57 | S2-FUV-2-60-08D-FU397V | 4,237 | 24 | 15 | 99 |
| S2 | 58 | S2-FUV-2-6-01F-FU111V | 2,635 | 18 | 43 | 130 |
| S2 | 59 | S2-FUV-2-62-08F-FU403V | 3,283 | 15 | 1 | 22 |
| S2 | 60 | S2-FUV-2-63-08G-FU406V | 10,000 | 27 | 7 | 62 |
| S2 | 61 | S2-FUV-2-68-09D-FU417V | 9,459 | 48 | 15 | 15 |
| S2 | 62 | S2-FUV-2-70-09F-FU424V | 6,899 | 26 | 5 | 96 |
| S2 | 63 | S2-FUV-2-7-01G-FU117V | 4,418 | 22 | 8 | 47 |
| S2 | 64 | S2-FUV-2-73-10A-FU433V | 8,047 | 45 | 8 | 26 |
| S2 | 65 | S2-FUV-2-74-10B-FU434V | 10,000 | 60 | 35 | 76 |
| S2 | 66 | S2-FUV-2-77-10E-FU439V | 8,450 | 31 | 12 | 15 |
| S2 | 67 | S2-FUV-2-79-10G-FU451V | 3,974 | 13 | 2 | 37 |
| S2 | 68 | S2-FUV-2-8-01H-FU123V | 3,868 | 26 | 15 | 80 |
| S2 | 69 | S2-FUV-2-81-11A-FU463V | 7,124 | 32 | 6 | 28 |
| S2 | 70 | S2-FUV-2-9-02A-FU126V | 3,426 | 13 | 2 | 42 |
| S3 | 71 | S3-FUV-2-10-02B-FU137V | 4,424 | 15 | 28 | 178 |
| S3 | 72 | S3-FUV-2-13-02E-FU152V | 7,288 | 28 | 12 | 51 |
| S3 | 73 | S3-FUV-2-17-03A-FU178V | 9,163 | 46 | 20 | 89 |
| S3 | 74 | S3-FUV-2-18-03B-FU191V | 7,272 | 33 | 6 | 55 |
| S3 | 75 | S3-FUV-2-2-01B-FU098V | 6,356 | 32 | 2 | 45 |
| S3 | 76 | S3-FUV-2-35-05C-FU254V | 10,000 | 36 | 6 | 190 |
| S3 | 77 | S3-FUV-2-39-05G-FU274V | 10,000 | 47 | 2 | 3 |
| S3 | 78 | S3-FUV-2-42-06B-FU283V | 7,018 | 24 | 11 | 78 |
| S3 | 79 | S3-FUV-2-43-06C-FU298V | 3,556 | 16 | 14 | 60 |
| S3 | 80 | S3-FUV-2-50-07B-FU339V | 3,006 | 7 | 3 | 44 |
| S3 | 81 | S3-FUV-2-53-07E-FU356V | 3,104 | 12 | 1 | 26 |
| S3 | 82 | S3-FUV-2-59-08C-FU396V | 10,000 | 58 | 10 | 150 |
| S3 | 83 | S3-FUV-2-80-10H-FU456V | 6,349 | 25 | 21 | 58 |

**Supplementary R Script**

library(randomForest)

rf=read.csv("Supplementary_Table_S4.csv", header=T)

rf_as=as.matrix(rf)

rf_num=data.matrix(rf)

features<-rf_num[,3:ncol(rf_num)]

labels<-rf_as[,1]

labels<-as.factor(labels)

classifier<-randomForest(x=features,y=labels,importance=T)

write.table(importance(classifier),"original_file_Supplementary_Table_S5.csv", col.names=T, sep=",")
